# T-Tubular Electrical Defects Contribute to Blunted β-Adrenergic Response in Heart Failure

**DOI:** 10.3390/ijms17091471

**Published:** 2016-09-03

**Authors:** Claudia Crocini, Raffaele Coppini, Cecilia Ferrantini, Ping Yan, Leslie M. Loew, Corrado Poggesi, Elisabetta Cerbai, Francesco S. Pavone, Leonardo Sacconi

**Affiliations:** 1 European Laboratory for Non-Linear Spectroscopy, Florence 50019, Italy; francesco.pavone@unifi.it (F.S.P.); sacconi@lens.unifi.it (L.S.); 2 National Institute of Optics, National Research Council, Florence 50125, Italy; 3 Division of Pharmacology, Department “NeuroFarBa”, University of Florence, Florence 50139, Italy; raffaele.coppini@unifi.it (R.C.); elisabetta.cerbai@unifi.it (E.C.); 4 Division of Physiology, Department of Experimental and Clinical Medicine, University of Florence, Florence 50134, Italy; cecilia.ferrantini@unifi.it (C.F.); corrado.poggesi@unifi.it (C.P.); 5 R. D. Berlin Center for Cell Analysis and Modeling, University of Connecticut Health Center, Farmington, CT 06030, USA; pyan@uchc.edu (P.Y.); les@uchc.edu (L.M.L.); 6 Department of Physics and Astronomy, University of Florence, Sesto Fiorentino 50019, Italy

**Keywords:** heart failure, T-tubules, excitation-contraction coupling, β-adrenergic signalling, non-linear microscopy imaging

## Abstract

Alterations of the β-adrenergic signalling, structural remodelling, and electrical failure of T-tubules are hallmarks of heart failure (HF). Here, we assess the effect of β-adrenoceptor activation on local Ca^2+^ release in electrically coupled and uncoupled T-tubules in ventricular myocytes from HF rats. We employ an ultrafast random access multi-photon (RAMP) microscope to simultaneously record action potentials and Ca^2+^ transients from multiple T-tubules in ventricular cardiomyocytes from a HF rat model of coronary ligation compared to sham-operated rats as a control. We confirmed that β-adrenergic stimulation increases the frequency of Ca^2+^ sparks, reduces Ca^2+^ transient variability, and hastens the decay of Ca^2+^ transients: all these effects are similarly exerted by β-adrenergic stimulation in control and HF cardiomyocytes. Conversely, β-adrenergic stimulation in HF cells accelerates a Ca^2+^ rise exclusively in the proximity of T-tubules that regularly conduct the action potential. The delayed Ca^2+^ rise found at T-tubules that fail to conduct the action potential is instead not affected by β-adrenergic signalling. Taken together, these findings indicate that HF cells globally respond to β-adrenergic stimulation, except at T-tubules that fail to conduct action potentials, where the blunted effect of the β-adrenergic signalling may be directly caused by the lack of electrical activity.

## 1. Introduction

In cardiomyocytes, stimulation of β-adrenergic receptors (β-AR) activates a powerful positive inotropic response via cAMP-dependent protein kinase A (PKA). PKA phosphorylates several key proteins that modulate cardiac function: L-type Ca^2+^ channels (LTCC) [1], ryanodine receptors (RyR2) [2], phospholamban [3], troponin I [4], and myosin-binding protein-C [5]. In heart failure (HF), one of the first causes of death worldwide [6], the β-AR system is compromised both in human and animal models [7,8,9,10], namely due to downregulation of mRNA and protein levels of β1-AR [11,12], one of the two main receptor isoforms that are present in the heart [13]. Moreover, HF is characterized by a plethora of (mal)adaptive mechanisms leading to myocardial remodelling [14]. Among those, HF distinguishes for ultrastructural and functional alterations of the transverse-axial tubular system (T-tubules), the complex sarcolemma invagination network that synchronously triggers action potentials (AP) in cardiac cells [15]. The two main isoforms of β-adrenergic receptors (β1 and β2-ARs) are both expressed on the T-tubular membrane and exert their function on the local excitation-contraction (EC) coupling machinery via cAMP and PKA [16]. However, the relative contribution of the two maladaptive processes, i.e., impairment of β-AR signalling and T-tubular defects, to the altered cardiomyocyte response to catecholamines remains undetermined.

Furthermore, beyond the alteration and loss of the T-tubular structure [17,18] observed in pathological settings, we recently demonstrated that electrical defects can occur even though the T-tubule structure is maintained [19], and cause compromised Ca^2+^ release [20]. With the present work, we assess the subcellular local Ca^2+^ release after acute β-AR stimulation in HF, in light of the new findings demonstrating the presence of T-tubular electrical defects that directly impair local Ca^2+^ release [20].

## 2. Results

### 2.1. Action Potentials and Ca^2+^ Transients in Failing Cells Treated with Isoproterenol

**Figure 1:**
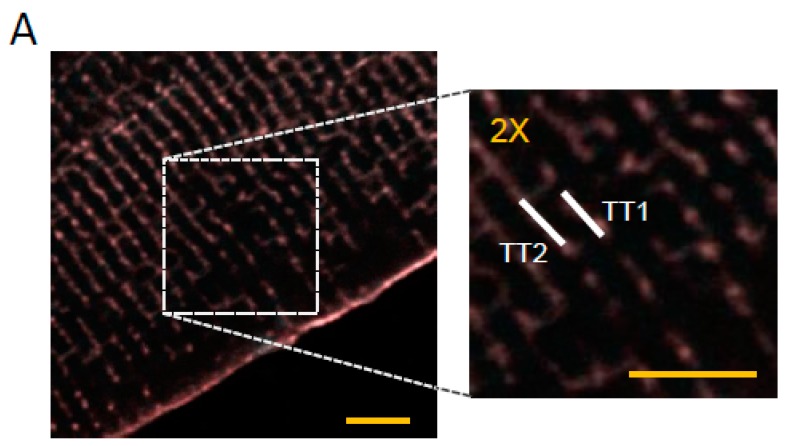

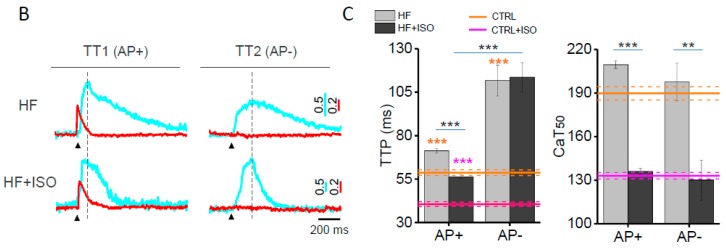
Ca^2+^ transient in HF cells treated with isoproterenol. (**A**) Two-photon fluorescence (TPF) image of a stained rat failing ventricular myocyte: sarcolemma in red (di-4-ANE(F)PTEA) and [Ca^2+^]_i_ in cyan (GFP-certified Fluoforte). Scale bar: 5 μm. On the right, a two-fold magnification of the region in the white dashed box. The lines mark the probed T-tubule (TT) sites: TT1 and TT2; (**B**) Average of 10 subsequent fluorescence traces (ΔF/F_0_) recorded from two scanned sites in a heart failure (HF) cell and in an isoproterenol-treated HF cell (HF + ISO, 10–7 M). AP is elicited at 200 ms (black arrowheads). Membrane voltage (red) and [Ca^2+^]_i_ (cyan); (**C**) Graphs showing Ca^2+^ release time-to-peak (TTP) and 50% decay (CaT50) in HF and HF + ISO cells. The failing TTs (AP−) have been distinguished from the electrically responsive ones (AP+). Data from 86 HF cells (506 AP+ and 23 AP−, *n* = 9), and from 55 HF + ISO cells (265 AP+ and 15 AP−, *n* = 9). Asterisks indicate significant differences (Student’s *t*-test, ** *p* < 0.01, *** *p* < 0.001). Ochre and magenta lines represent the Ca^2+^ kinetics features measured nearby TTs of, respectively, CTRL and of isoproterenol-treated CTRL (CTRL + ISO) cells: mean (solid) ± SE (dashed). CTRL and CTRL + ISO data from previously published data [20]. Ochre and magenta asterisks refer to the comparison with CTRL or CTRL + ISO values, respectively.

We previously demonstrated [19] that the HF rat model is characterized by the co-presence of electrically uncoupled and electrically coupled T-tubules, named AP− and AP+, respectively. This electrical defect profoundly impacts the local Ca^2+^ release [20]. Here, we find that isoproterenol does not restore the function of uncoupled T-tubules. In fact, HF cells acutely exposed to 10^−7^ M isoproterenol show 5.6% ± 3% of AP− (*n* = 265 from 55 cells, nine animals), in line with our previous findings in HF cells at basal conditions [19,20]. In Figure 1B, we report representative voltage traces (in red) and the corresponding Ca^2+^ transients (in cyan) recorded from AP+ and AP− T-tubules in the absence (Figure 1A, HF) and presence of isoproterenol (HF + ISO). The effect of β-AR stimulation on the Ca^2+^ rise in correspondence to AP+ tubules is qualitatively different from that observed in AP− elements. In fact, the expected reduction of Ca^2+^ transient time-to-peak (TTP) after isoproterenol is observed exclusively in the proximity of AP+, while Ca^2+^ transient TTP of AP− is not affected by isoproterenol treatment (Figure 1C). On the contrary, the 50% decay of Ca^2+^ transients (CaT50) is significantly hastened in HF + ISO cardiomyocytes as compared with HF cells at basal condition at both AP+ and AP− sites. These results suggest that the Ca^2+^ transient detected at AP− tubules results from the Ca^2+^ signal propagating from neighbouring sites, and the velocity of propagation is not regulated by β-AR signalling. Contrarily, the increased phosphorylation of phospholamban by PKA activation is preserved, increasing the sarcoplasmic reticulum Ca^2+^ ATPase (SERCA) reuptake rate across the whole cardiomyocyte, at both AP+ and AP− sites.

### 2.2. Spatio-Temporal Variability of Ca^2+^ Transients in Isoproterenol-Treated Failing Cardiomyocytes

**Figure 2:**
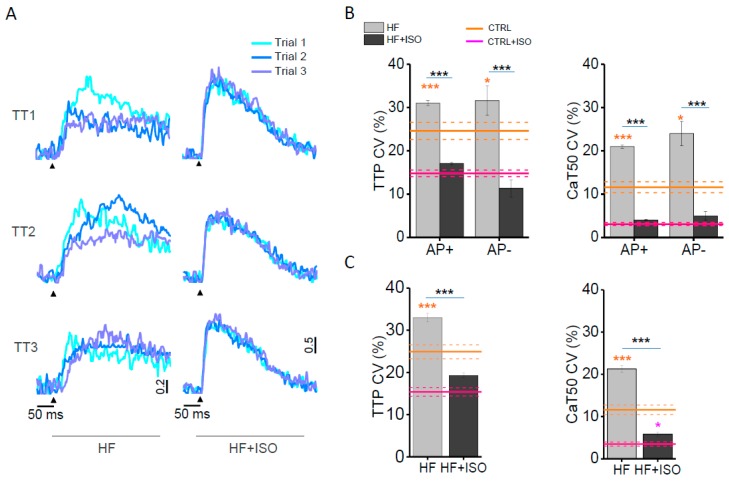
Spatio-temporal variability of Ca^2+^ transient in HF cells treated with isoproterenol. (**A**) Superposition of three subsequent Ca^2+^ transients recorded in three different T-tubules (TTi) of HF and HF + ISO cardiomyocytes; (**B**,**C**) Graphs showing Ca^2+^ release coefficient of variability (CV) calculated at time-to-peak (TTP) and 50% Ca^2+^ transient decay (CaT50) based on time (beat-to-beat CV) and on space (spatial CV). AP+ and AP− HF and HF + ISO are separately analysed in beat-to-beat CV. Asterisks indicate significant differences (Student’s *t*-test, * *p* < 0.05, *** *p* < 0.001). Data from 86 HF cells (506 AP+ and 23 AP−, *n* = 9), and from 55 HF + ISO cells (265 AP+ and 15 AP−, *n* = 9). Ochre and magenta lines represent the Ca^2+^ transient CV features measured nearby TTs of, respectively, CTRL and of isoproterenol-treated CTRL (CTRL + ISO): mean (solid) ± SE (dashed). CTRL data from previously published data [20]. Ochre and magenta asterisks refer to the comparison with CTRL or CTRL + ISO values, respectively.

By studying multiple T-tubule sites within the same cell, we can assess the variability of Ca^2+^ transients both in time (beat-to-beat; at the same site) and in space (among different sites). A coefficient of variability (CV) is calculated as σ/μ, where σ is the standard deviation and μ is the mean. The CV of Ca^2+^ release is calculated based on time (beat-to-beat CV) and space (spatial CV) and the corresponding graphs are reported in Figure 2B,C. We previously demonstrated that even in CTRL cardiomyocytes, T-tubules display a non-negligible beat-to-beat and spatial variability of the rate of Ca^2+^ release; such variability is significantly reduced with isoproterenol application [20]. In HF cells, the CV of TTP is significantly higher than that of the CTRL in the vicinity of both AP+ and AP−. Ca^2+^ decay of failing cardiomyocytes is also more variable when compared to CTRL cells. In Figure 2A, we superimposed three subsequent Ca^2+^ traces recorded in three different T-tubules of a HF and HF + ISO myocyte. It can be noticed that isoproterenol application reduces the variability of local Ca^2+^ release and reuptake in HF cells and, as reported in the columns (Figure 2B,C), the variability of every parameter in HF + ISO is similar to that observed in CTRL + ISO cardiomyocytes. The synchronizing effect of β-AR treatment on Ca^2+^ transients is likely due to enhanced RyR2 recruitment by increased channel phosphorylation [21].

### 2.3. Isoproterenol Effect on Ca^2+^ Sparks Frequency in Heart Failure

**Figure 3:**
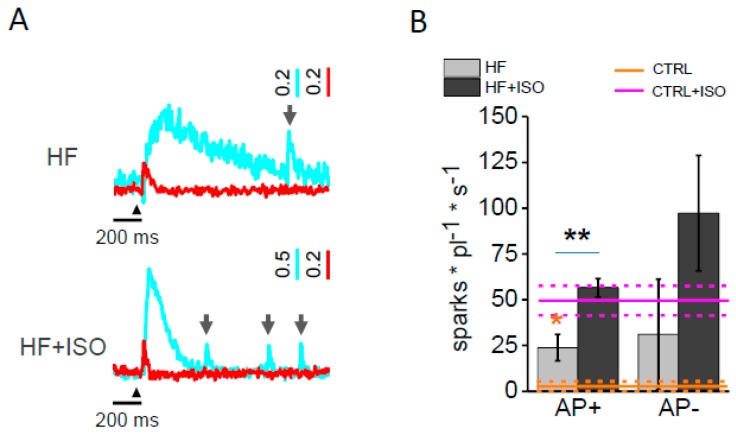
Ca^2+^ sparks in HF cells treated with isoproterenol. (**A**) Two representative fluorescence traces (ΔF/F0) recorded from HF and HF + ISO cells. The grey arrow pinpoints Ca^2+^ sparks occurrence; (**B**) Columns showing mean Ca^2+^ sparks frequency (fs) recorded in AP+ and AP− from HF and HF + ISO myocytes. Asterisks indicate significant differences (Student’s *t*-test, * *p* < 0.05 ** *p* < 0.01). Data from 86 HF cells (506 AP+ and 23 AP−, *n* = 9), and from 55 HF + ISO cells (265 AP+ and 15 AP−, *n* = 9). Ochre and magenta lines represent the (fs) measured in CTRL and of isoproterenol-treated CTRL (CTRL + ISO), respectively: mean (solid) ± SE (dashed). CTRL data from previously published data [20]. Ochre and magenta asterisks refer to the comparison with CTRL or CTRL + ISO values, respectively.

To further evaluate β-AR’s role on RyR open probability (Po), we studied the Ca^2+^ spark frequency. Ca^2+^ sparks are defined as spontaneous Ca^2+^ release events occurring at a single Ca^2+^ release unit (CRU) [22] during a regularly paced sequence of Ca^2+^ transients. Here, we confirmed that the Ca^2+^ sparks frequency during diastole is dramatically increased after isoproterenol treatment in control cardiomyocytes, their frequency being 1.8 ± 1.8 mHz and 32.9 ± 5.4 mHz, without and with isoproterenol treatment, respectively. In Figure 3A, we reported two representative fluorescence traces showing the occurrence of Ca^2+^ sparks (grey arrow) in HF (above) and in isoproterenol-treated HF cardiomyocytes (bottom). In HF, we previously reported a significantly augmented Ca^2+^ sparks frequency in isolated cardiomyocytes [20] as compared to the control. Our result did not depend on the electrical activity of the corresponding T-tubule. In fact, electrically coupled T-tubules and failing elements displayed similarly increased levels of Ca^2+^ sparks in their vicinity. Here, the isoproterenol treatment of HF cardiomyocytes significantly enhances the frequency of Ca^2+^ sparks in AP+ (Figure 3C) as compared to CTRL cells treated with isoproterenol. In line with previous results [20], AP− elements show the same tendency after isoproterenol treatment. This result suggests that HF cells respond to β-adrenergic signalling with an augmented Ca^2+^ leakage from the sarcoplasmic reticulum (SR) similarly to CTRL cells, irrespective of T-tubule excitability.

## 3. Discussion

In the present work, we employ an optical technique capable of simultaneously studying Ca^2+^ and voltage at a sub-cellular level, in order to disclose the effects of β-AR signalling on Ca^2+^ release at the local level in failing rat cardiomyocytes. As demonstrated by our investigations on Ca^2+^ sparks and Ca^2+^ transient variability, we find that acute stimulation of the β-AR system increases the open probability (Po) of RyR channels by a similar extent in HF cells and control cardiomyocytes. It is of note that the RAMP microscope has a sensitivity capable of detecting single Ca^2+^ sparks [20], by probing a volume that contains about 5–10 CRUs (~10 μm^3^) [23,24]. In our work, Ca^2+^ sparks are not evoked by cellular permeabilization or specifically designed stimulation protocols, but occur spontaneously in intact cardiomyocytes during regular pacing at steady state. We confirmed that stimulation of β-AR signalling increases Ca^2+^ sparks frequency [25] in intact control cardiomyocytes. In HF, β-AR signalling is disrupted, leading to an excessive phosphorylation of RyR [2] and decreased binding of RyR to its regulatory protein FKBP12.6 [26]. These modifications are, however, accompanied by decreased activity of SERCA as well as increased activity of NCX, eventually determining reduced SR load. In such a scenario, it is complicated to predict whether the Ca^2+^ sparks rate is changed in HF compared to control cells. Previous work by Gomez et al. did not observe an increased frequency of Ca^2+^ sparks in failing rat cardiomyocytes [27], while an augmented SR Ca^2+^ leak has been found in intact ventricular myocytes of failing rabbits [28]. In the present work, Ca^2+^ sparks are increased compared to the control in the absence of β-AR stimulation. The factors influencing RyR gating have been extensively reviewed [29,30], the major culprits being probably the RyR phosphorylation performed by the calcium-calmodulin–dependent protein kinase (CaMKII) [31] and other post-translational modifications of RyR2 found in HF [32,33]. PKA-dependent phosphorylation of RyR does not seem to increase the RyR leak [34]. After β-AR stimulation, we found that the Ca^2+^ sparks frequency is increased in HF cardiomyocytes similarly to isoproterenol-treated control cells. This finding suggests that β-AR signalling still has leeway to generate Ca^2+^ sparks in HF cells, using the same mechanisms of control cells (i.e., increased RyR2 open probability and SR Ca^2+^ load). In addition, the microenvironment pertinent to failing T-tubules shows a Ca^2+^ sparks rate comparable to that of electrically coupled elements and displays a similar response to β-AR stimulation.

Moreover, we observe a synchronization of RyR2 recruitment upon β-AR challenge as demonstrated by the coefficient of variability of Ca^2+^ transients. In fact, a homogeneous recruitment of RyR2 operated by β-AR signalling is observed both in space and in time and it is similar in failing and CTRL cardiomyocytes after isoproterenol challenge. Altogether, these observations allow inferring that, despite altered basal levels, the β-AR signalling on RyR2 is well preserved in HF cells.

Alterations of Ca^2+^ release have been previously observed in HF cells with structurally remodelled T-tubules [35,36] and wall stress progressively determines the level of structural disruption and the consequences on Ca^2+^ release [37]. Although the lack of T-tubules represents a perturbation of Ca^2+^ handling per se, here we focused on the microenvironment around preserved T-tubules. Employing a novel functional imaging technique [38], we have demonstrated that about 6% of T-tubules fail to propagate AP in HF [19,20] and the entity of this defect can be much larger in other pathologies, e.g., 22.7% ± 5% in mice carrying a mutation associated with hypertrophic cardiomyopathy [39]. Such T-tubular electrical failure exerts a major impact on the Ca^2+^ rise in HF [20] which has to be added to the abnormal Ca^2+^ release previously observed. In HF cardiomyocytes, the AP failure prevents LTCC activation, because, even though β-AR signalling phosphorylates LTCC, this response does not translate into higher sarcolemmal Ca^2+^ influx or into the downstream SR Ca^2+^ release. In fact, shortening of the Ca^2+^ transient TTP is reduced by β-AR activation exclusively in the vicinity of electrically coupled T-tubules (AP+). The acceleration of Ca^2+^ transient TTP after β-AR stimulation in HF cells is lower than in control cardiomyocytes. This can be explained by the fact that HF cardiomyocytes show a lower density of LTCC but a higher level of basal Ca^2+^ channel phosphorylation [40], likely limiting the ability of β-AR activation to further augment LTCC. In addition, β-AR stimulation in HF has been associated with the impaired formation of E-C coupling microdomains [41] that may further reduce the β-AR signalling role on LTCC. It is of note that β-AR stimulation speeds up Ca^2+^ transient decay at every location in HF cells. It means that β-AR–mediated phosphorylation of phospholamban is maintained in the cardiomyocyte, increasing the SERCA reuptake rate across the whole cell. Again, this is the hint that β-AR machinery is still capable of working in failing cardiomyocytes when the target substrates are present. 

In conclusion, our work provides functional information about the subcellular responsiveness to β-AR signalling in HF that should be integrated in the complex scenario of the β-adrenergic molecular modifications found in this disease. Here, we provide evidence that a novel mechanism for the blunted β-AR signalling is present in HF and it does not directly involve receptors and downstream effectors, but it is caused by the absence of electrical activity in some T-tubules.

## 4. Materials and Methods

### 4.1. Cardiomyocyte Preparations and Labelling

Ventricular myocytes were isolated from male Wistar Han rats (300–350 g, Harlan Laboratories SRL) as previously described [19]. Myocardial infarction is induced by ligation of the left anterior coronary artery as previously described [18]. In this class of experiments male Wistar Han rats (190–230 g, Harlan Laboratories SRL) were used. Cardiac function was monitored with echocardiography before surgery and was periodically checked after the intervention. Six weeks after the infarction, a left ventricular dilatation occurs, together with a loss of contractile function. Echocardiographic data have been previously reported [20]. In details, we measured the end-diastolic diameter (EDD) and the end-systolic diameter (ESD) in four CTRL and 18 HF rats. We found that the left-ventricular fractional shortening calculated as (EDD–ESD)/EDD in percentage is significantly decrease. Moreover, HF rats showed arrhythmias during the echocardiographic procedure that have never been observed in CTRL. Student’s *t*-test applied. Rats were sacrificed six to eight weeks after surgery and used for cell isolation. All animal procedures are performed conform the guidelines from Directive 2010/63/EU of the European Parliament on the protection of animals used for scientific purposes; experimental protocol is approved by the Italian Ministry of Health on the 6th of July 2015 (approved protocol number 647/2015-PR). Cells were loaded in extracellular buffer added with 10 μM blebbistatin, 4 μM cytochalasin D, and 500 μM CaCl_2_. First, 0.5 μg/mL of GFP-certified Fluoforte dissolved in DMSO were added to the cell suspension for 15 min. After washing, 2 μg/mL of di-4-AN(F)EPPTEA dissolved in ethanol was also added for 15 min and then cells were resuspended in fresh extracellular buffer containing 10 μM blebbistatin, 4 μM cytochalasin D and 1 mM CaCl_2_. Loaded preparations were used for experiments within 1 h. Although blebbistatin is known to be fairly safe [42], cytochalasin D was found to exert a slight effect on Ca^2+^ transient at 40 μM [43]. For this reason, to avoid any artifact in measurements, we applied cytochalasin D in all experimental groups at a quite low concentration. The staining and imaging session were performed at room temperature (20 °C).

### 4.2. RAMP Microscope and Optical Recording

The basic design of our RAMP imaging system has been already described [20]. Briefly, it consists of a 1064 nm fiber laser, an acousto-optic modulator for angular spreading pre-compensation and two orthogonally mounted acousto-optic deflectors for laser scanning. The fluorescence signal was collected in backward direction by the excitation oil immersion objective (63 × NA 1.4; Zeiss) and in forward direction using a high numerical aperture condenser lens. For each detection direction, a dichroic mirror was used to split the two spectral components of the fluorescence signal, the red and the green emission light. The fluorescence signal was detected by two independent photon counting modules based on GaAsP photomultiplier tube (H7422, Hamamatsu). Emission filters of 655 ± 20 nm and 520 ± 16 nm were used for voltage and Ca^2+^ detection, respectively. The measurements were performed during steady-state stimulation (0.34 Hz). The cells were field-stimulated using two parallel platinum wires (250 μm in diameter) placed at a distance of 6.3 mm. Square pulses of 10–20 V and duration of 3 ms were used to reach AP threshold. In a typical measurement, we probed 5–10 different sarcolemmal sites for ten subsequent trials. The length of the scanned lines ranged from 2 to 10 μm with an integration time per membrane pass of ~200 μs, leading to a temporal resolution of 0.4–2 ms. The large Stokes shift of fluorinated VSD is not sufficient to prevent spectral contamination between the two channels. For this reason, we optimized a simple un-mixing procedure under two hypotheses: negligible contamination of the green channel on the red one and constant VSD sensitivity across the emission spectrum.

### 4.3. Data Analysis and Statistics

Optical data were analysed with software written in LabVIEW 2010 (National Instruments). The amplitude and kinetics parameters of the Ca^2+^ were manually identified trace by trace for the calculation of the coefficient of variation (CV), while the mean values of each probed site were determined after averaging 10 subsequent trials to increase accuracy. Spontaneous Ca^2+^ sparks were scored when a sudden increase of fluorescence intensity occurred with a ΔF/F_0_ two-fold above the trace noise not correlated to the electrical stimulus. Ca^2+^ spark frequency was normalized to time and excitation volume (sparks·pl^−1^·s^−1^), assuming the two-photon excitation volume as $V_{TPE}=\pi^{3/2}\omega_{x}\omega_{y}\omega_{z}/0.68$, where (ω) is 1/e widths of the lateral (*xy*) and axial (*z*) intensity squared profiles [44]. An average length of 6 μm is used for the scanned lines. VSD sensitivity was estimated based on the evidence that AP amplitude of 100 mV corresponds to a fluorescence variation of 20%. The T-tubules showing failures were scored using threshold ΔF/F_0_ = 0.037 in agreement with our previous finding [19]. In order to analyse diastolic Ca^2+^ sparks, we calculated the systole as the CaT95 for each experimental group, the rest of the recording is considered diastole. Data are expressed and plotted as means ± SEM (Standard Error of Mean) obtained from a number of independent determinations on different myocytes. Number of cells and number of animals (N) are indicated in the figure legends for each set of measurements. Unpaired Student’s *t*-test is used for comparisons. A *p*-value of < 0.05 is considered statistically significant (* *p* < 0.05, ** *p* < 0.01, *** *p* < 0.001).
